# Coping With Anxiety: Brain Structural Correlates of Vigilance and Cognitive Avoidance

**DOI:** 10.3389/fpsyt.2022.869367

**Published:** 2022-04-07

**Authors:** Vivien Günther, Salome Jahn, Carolin Webelhorst, Charlott Maria Bodenschatz, Anna Bujanow, Simone Mucha, Anette Kersting, Karl-Titus Hoffmann, Boris Egloff, Donald Lobsien, Thomas Suslow

**Affiliations:** ^1^Department of Psychosomatic Medicine and Psychotherapy, University of Leipzig Medical Center, Leipzig, Germany; ^2^Department of Neuroradiology, University of Leipzig Medical Center, Leipzig, Germany; ^3^Department of Psychology, Johannes Gutenberg University of Mainz, Mainz, Germany

**Keywords:** avoidant coping, vigilant coping, repression, sensitization, MRI, voxel-based morphometry

## Abstract

**Background:**

Individuals differ in their dispositional coping behavior when they are confronted with anxiety-provoking situations. Cognitive avoidance is characterized by a withdrawal from threatening information, whereas vigilance denotes the intensive search for threat-related information. Functional neuroimaging studies indicate alterations in brain responsivity to emotional stimuli as a function of cognitive avoidant and vigilant coping, but findings are partially discrepant. Studies on structural correlates of coping styles are scarce.

**Materials and Methods:**

By using structural magnetic resonance imaging, the present study examined the relationship between brain gray matter volume and coping strategies in 114 healthy individuals. Individual differences in vigilance and cognitive avoidance were measured by the Mainz Coping Inventory.

**Results:**

Exploratory whole-brain analyses were conducted. Cognitive avoidant coping significantly predicted reduced gray matter volume in the bilateral thalamus, whereas vigilant coping was associated with volumetric increases in the bilateral thalamus. These relationships remained significant when controlling for a potential influence of age, sex, depressive symptoms, and trait anxiety.

**Discussion:**

Our findings indicate that dispositional strategies to deal with anxiety-provoking situations are related to volumetric alterations in the thalamus, a brain structure that has been implicated in the mediation of attentional processes and alertness, and the anticipation of harm. The dispositional tendency to monitor the environment for potential threats (i.e., vigilance), appears to be associated with volumetric increases in the thalamus, whereas the dispositional inclination to divert one’s attention away from distressing stimuli (i.e., cognitive avoidance) seems to go along with reductions in thalamic gray matter density.

## Introduction

Anxiety is defined as an emotional condition that is characterized by feelings of distress and apprehension, which are accompanied by increased physiological activity (1). Individuals differ in their dispositional preferences for cognitive coping strategies when confronted with anxiety-provoking situations. In Krohne’s (2) model of coping modes (MCM), two dimensions of dispositional coping behavior in threatening situations have been suggested: cognitive avoidance and vigilance. Cognitive avoidance is characterized by the inhibition of the processing of threatening information. This aim is achieved by directing attention away from internal or external threatening cues. In contrast, vigilance denotes the disposition to intensely search for threat-related information under stressful conditions to prevent the possible occurrence of bad surprises. These coping concepts are based on the assumption that most anxiety-evoking events are determined by the presence of aversive stimulation and ambiguity. In turn, these situational features trigger experiences of emotional arousal and uncertainty concerning the expectancy of threat. Whereas, cognitive avoidance is assumed to reduce intense anxiety states by shielding the individual from aversive stimulation, vigilance serves the purpose of reducing uncertainty and minimizing the probability of unexpected negative events. According to the MCM, individuals with a low tolerance for uncertainty are supposed to employ a vigilant coping style, accompanied by an intense monitoring of their environment in search for potentially threatening signals, whereas individuals highly susceptible for anxious arousal should tend to avoid danger cues. Both dimensions of dispositional coping are assumed to be relatively independent from each other [see also (3)]. Although cognitive avoidance and vigilance appear to be moderately correlated with each other, factor analyses provided convincing evidence for a two-dimensional conceptualization (4, 5). Individuals scoring high in avoidance, but low in vigilance, are often designated as repressors, whereas individuals who consistently employ vigilance, but not avoidance, are conceptualized as sensitizers [e.g., (4)]. According to Weinberger et al.’s (6) theory of coping strategies, repressors avoid the awareness of their own anxiety, and score low in self-reported anxiety, while they often display higher physiological responses to stress. Hence, repression was defined by low trait anxiety, paired with high repressive defensiveness. Repressors appear to deny negative information regarding the self, including their own negative affect [see (7) for an overview]. Hence, to classify repression, Weinberger et al. (6) have suggested the simultaneous use of trait anxiety scales along with social desirability scales. A considerable overlap between Krohne’s (2) and Weinberger et al.’s (6) coping style classifications have been observed (4, 8), indicating that both methods assess related constructs, particularly with respect to repression and sensitization. Indeed, there is evidence that cognitive avoidance is associated with lower levels of trait anxiety and neuroticism, whereas vigilance shows a positive correlation with anxiety and neuroticism (4, 9–12). However, relations among vigilance, cognitive avoidance and social desirability appear rather weak (5, 8, 10). Despite some differences in their theoretical foundations, findings based on Weinberger et al.’s (6) and Krohne’s (2) assessment procedures appear comparable (8). Both methods were able to reveal in avoidant copers and repressors discrepancies among low self-reported distress and high levels of physiological arousal in response to a stressor [e.g., (13–15)].

Regarding the functional and structural neural correlates of avoidant and vigilant coping styles, as conceptualized by Krohne (2), neuroimaging studies are scarce and have revealed partly mixed results. Although cognitive avoidance and vigilance are not considered as opposite ends on the same continuum of a trait variable, most studies that investigated threat processing have compared repressors to sensitizers. Rauch et al. (16) applied a passive viewing task with briefly displayed and clearly visible threatening faces. Repressors, relative to sensitizers, have demonstrated increased activations in areas involved in attentional control and emotion regulation [i.e., anterior cingulate gyrus and ventromedial prefrontal cortex; e.g., see (17)] and in the occipito-temporal visual system in response to fearful faces. On the other hand, heightened responsivity to angry faces in ventromedial and ventrolateral prefrontal areas, and in the amygdala was found in sensitizers. During an emotion recognition task with very briefly presented emotional faces, repressors have shown stronger prefrontal, temporal and parietal activations in response to both angry and fearful faces, relative to sensitizers (18). According to the authors, these stronger activations may indicate a potential hypersensitivity and an intensified processing of threatening stimuli on early information processing stages in repressors, but may also represent heightened emotion regulatory efforts and attentional control, particularly in prefrontal areas [see also (16, 19)]. Similar results have been reported by Rauch et al. (19) for an emotion-evaluation task. Here, repressors (relative to sensitizers) exhibited exaggerated responsiveness to angry, but not fearful faces, in frontal, temporal and parietal areas. When considering activations triggered by neutral faces, relative to angry faces, sensitizers appear to demonstrate heightened frontal, temporo-parietal, occipital as well as hippocampal and thalamic responsivity. Neutral faces may be considered as more ambiguous than angry faces with regard to their threat value. Therefore, Rauch et al. (19) have argued that sensitizers might process these stimuli more intensively, probably in an attempt to disambiguate their meaning.

During a fear conditioning procedure with electrical stimulation (20), repressors have responded stronger in emotion related brain areas (i.e., the amygdala and insula), but also in the ventromedial prefrontal cortex and anterior cingulate gyrus, thus, brain areas that are associated with the down-regulation of emotions.

Interestingly, Leehr et al. (21) implemented an implicit emotion processing task in a large sample and used a regression approach to predict brain responsiveness to threatening faces by vigilant and avoidant coping styles, each independently. Here, no associations between cognitive avoidance and neural responses to angry and fearful faces were revealed. Only vigilance appeared to be related to decreased responsiveness in the anterior cingulate gyrus to angry faces, but not fearful faces. Moreover, no relationships between coping styles and density of gray matter (GM) were observed. To our knowledge, Leehr et al. (21) conducted the only study in which structural correlates of vigilance and cognitive avoidance were investigated.

In sum, when comparing repressors with sensitizer, several studies point to hyper-activations in prefrontal, temporal, and parietal areas. Increased activations in repression may be possible indicators for enhanced processing of threat, or for increased emotion regulation efforts. However, findings appear to strongly depend on the emotional quality of threat stimuli, with angry faces inducing more robust activations than fearful faces. Although repressors and sensitizers are known to differ in their trait anxiety, none of these studies has taken a potential influence of this variable into account. One study, using a dimensional approach in a large sample, could not confirm an association between cognitive avoidance and cerebral hyper-responsiveness (21). Regarding a vigilant coping style, previous results are even more mixed. Thus, findings remain heterogeneous and point out the importance of further research. For the purpose of meta-analyses, more studies that investigate functional or structural alterations as a function of coping styles are required.

In the present study, structural magnetic resonance imaging scans were obtained from healthy adults with varying degrees of cognitive avoidant and vigilant coping strategies. We aimed to examine the relationship between the dispositional preference for vigilant/avoidant coping styles and GM volumes in an exploratory approach. Due to the discrepancy of previous results, no regions of interest were defined. Since coping strategies in anxiety-provoking situations are related to trait anxiety [e.g., (9)], we also investigated whether vigilance and cognitive avoidance could explain an incremental proportion of variance in GM density, relative to trait anxiety.

## Materials and Methods

### Participants

One hundred and eighteen healthy volunteers took part in this study. Four participants did not complete the study, thus our final sample included 114 participants (63 women). Participants were right-handed, native German speakers with a mean age of 25.53 years (*SD* = 3.46) and a mean school education of 12.40 years (*SD* = 0.56, range: 12–13). They were recruited *via* public notices that were posted in canteens, libraries and student halls of residence. A history of neurological or psychiatric diseases, intake of psychotropic medication, contraindications for magnetic resonance imaging, and head trauma involving loss of consciousness were exclusion criterions for study participation. The Structured Clinical Interview for DSM-IV Axis I disorders [SCID-I (22)] was administered to rule out diagnoses of current or past Axis I disorders.

The study was approved by the local ethics committee. After a detailed explanation of the study, written informed consent was obtained from all participants. The participants received a financial compensation of 40 Euro.

### Psychometric Measures

Vigilant and cognitive avoidant coping styles were assessed by the Mainz Coping Inventory [MCI (4, 9)]. The MCI is constructed as a stimulus-response inventory consisting of 80 items, which are rated on a true-false scale. The questionnaire encompasses the description of eight anxiety-evoking scenarios, including physical threat (e.g., flight turbulences or the encounter with a group of dubious people at night) and ego threat (e.g., giving a speech or making a mistake on the job). Five cognitive avoidant strategies, such as “attentional diversion,” “re-interpretation,” and “denial” (e.g., “I prefer to talk with friends about something other than the speech” or “I put on my headphones and listen to music”), and five vigilant strategies, such as “information search” and “anticipation of negative events” (e.g., “I think about what questions might be asked after the speech” or “I watch the dubious people closely”) are assigned to each threat situation. Participants are required to indicate which of the mentioned strategies they would employ in the respective scenario. Sum scores are calculated separately for the vigilance and cognitive avoidance items across all situations. In our sample, the internal consistency (Cronbach’s α) was satisfactory with α = 0.81 for the cognitive avoidance scale (MCI-CAV) and α = 0.80 for the vigilance scale (MCI-VIG). Levels of general anxiety were assessed with the trait version of the State-Trait Anxiety Inventory [STAI (23)]. The STAI-trait consists of 20 items evaluating feelings of tension, restlessness, insecurity, worry and dissatisfaction. The internal consistency was good in our sample (Cronbach’s α = 0.88). In addition, trait anxiety was assessed with the trait version of the Beck Anxiety Inventory [BAI-T (24)], a measure that should be less confounded with depression than the STAI. The BAI-T contains 21 items assessing the experience of anxious symptoms, such as palpitation and tenseness, according to their frequency of occurrence on a day-to-day basis. Internal consistency for the BAI-T in our sample was satisfactory (Cronbach’s α = 0.90). The Beck Depression Inventory II [BDI-II (25)] contains 21 items and was administered to assess level of depressive symptoms. The internal consistency of the BDI-II was good (Cronbach’s α = 0.83). Table 1 presents descriptive statistics and correlations between all psychometric measures. Of note, there were significant sex differences for cognitive avoidance, with men showing higher values than women [*t*(112) = 2.07; *p* = 0.04], see Table 1. No additional sex differences were observed (all *p*s > 0.14). Moreover, cognitive avoidant and vigilant coping strategies were moderately negatively related.

**TABLE 1:** Descriptive statistics and correlations between psychometric measures.

	All	Men	Women	Coping	Affectivity		
	*M* (*SD*)	*M* (*SD)*	*M* (*SD*)	MCI-VIG	STAI	BAI-T	BDI-II
MCI-CAV	23.19 (6.01)	24.47 (5.46)	22.16 (6.27)	–0.39*	–0.29*	–0.31*	–0.03
MCI-VIG	21.23 (5.99)	20.29 (6.45)	21.98 (5.53)	–	0.37*	0.35*	0.18
STAI	36.10 (7.89)	37.14 (6.89)	35.25 (8.58)	–	–	0.65*	0.43*
BAI-T	10.48 (8.29)	10.29 (7.22)	10.63 (9.12)	–	–	–	0.41*
BDI-II	5.07 (4.58)	5.65 (4.67)	4.60 (4.50)	–	–	–	–

*p < 0.01 (two-tailed).

### MRI Data Acquisition, Preprocessing, and Analyses

High-resolution structural MR images were obtained using a 3 T scanner (Magnetom Trio, Siemens, Erlangen, Germany). Structural images were acquired with a T1-weighted 3D MP-RAGE (26). The imaging parameters were TI 900 ms, TR 1,900 ms, TE 2.65 ms, flip angle 9°, spatial resolution of 0.8 × 0.8 × 1 mm^3^, two averages. The VBM8-toolbox^1^ was used for preprocessing the structural images with recommended default parameters. GM segments were modulated only by the non-linear components in order to preserve actual GM values locally. These procedures require no further correction for total brain volume in the statistical model, as the corrections is directly applied to the data. The preprocessed GM images were smoothed (Gaussian kernel size: 8 mm FWHM) and had a spatial resolution of 1.5 × 1.5 × 1.5 mm^3^.

The check-data-quality module was applied to test for homogeneity of GM images using the covariance structure of each image with the other images. None of the participants showed artifacts or anatomical abnormalities in the images.

Second-level analyses were performed using SPM12. The modulated GM images of participants were entered into two regression models with the respective individual MCI-CAV and MCI-VIG scores as regressor of interest. Exploratory whole-brain analyses were conducted with a voxel-wise threshold at *p* < 0.001 (uncorrected) and a cluster-level threshold of *p* < 0.05, family wise error (FWE) corrected. For the purpose of meta-analyses and for interested readers, results with a less stringent threshold (cluster-level threshold: *p* < 0.05, *un*corrected, including clusters > 162 voxels) are provided in Table 2.

**TABLE 2:** Brain regions showing alterations in GM volume as a function of coping styles at a cluster-level threshold of *p*_*uncorrected*_ < 0.05.

	Hemisphere	Peak *T*-value	Peak-level	Cluster size (voxels)	Cluster-level	Peak MNI		
			*p*_uncorrected_		*p*_uncorrected_	*x*	*y*	*z*
**Vigilance (positive correlations)**								
Thalamus	R/L	4.36	<0.001	1,523	<0.001	5	–18	13
Middle temporal gyrus	L	4.24	<0.001	357	0.007	–50	–16	–9
Hippocampus	R	3.62	<0.001	225	0.03	23	–36	4
**Cognitive avoidance (negative correlations)**								
Thalamus	R/L	3.59	<0.001	827	<0.001	–2	–15	–3
Superior parietal gyrus	R	4.76	<0.001	231	0.02	32	–60	49
Superior temporal gyrus	R	4.72	<0.001	197	0.03	60	–30	4

To investigate whether coping styles share an incremental proportion of variance with GM volume, relative to anxiety, additional two-stage hierarchical regression analyses were calculated in SPSS25. To do this, we extracted GM volume values, averaged across all voxels within a significant cluster for each participant [by means of the MarsBaR toolbox, see (27)] as dependent variables. In a first step, STAI, BAI-T, BDI-II, age, and sex were entered as regressors. In a second step, cognitive avoidant and vigilant coping styles were included as regressors, each, respectively.

Cognitive avoidance and vigilance were significantly (negatively) correlated. Thus, for brain areas, which were revealed for both coping styles, additional hierarchical regression analyses were carried out to evaluate the individual contribution of each scale to explained variance in the respective GM volume.

## Results

### Cognitive Avoidance

**FIGURE 1:**
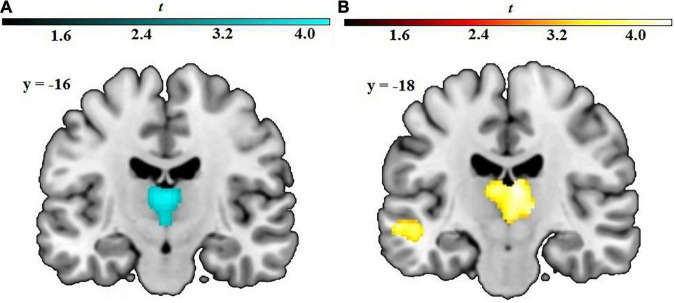
Results from whole-brain regression analyses with **(A)** cognitive avoidance and **(B)** vigilance predicting GM volume. Axial images in neurological orientation showing the relation among **(A)** high cognitive avoidance and decreased GM volume in the bilateral thalamus, and among **(B)** high vigilance and volumetric increases in the bilateral thalamus and left middle temporal gyrus. The voxel-wise threshold was set to *p* = 0.001 (uncorrected) with a cluster-level threshold of *p* < 0.05, FWE-corrected. Color bar: *t*-values.

Regression analyses yielded a significant cluster in the left and right thalamus (peak voxel xyz: –2 –15 –3, *T* = 3.70, *p* < 0.001, cluster size: 827, cluster *p*_FWE_ = 0.002), indicating a negative correlation between cognitive avoidance and GM volume, see Figure 1A.

In the first step of hierarchical regression analyses in SPSS, variance in thalamic GM volume was significantly explained by sex (β = 0.36; *p* < 0.001) and BDI-II (β = –0.19; *p* <= 0.05), but not by STAI, BAI-T, or age [*p*s > 0.13, *R*^2^ = 0.22; *F*(5, 108) = 6.10, *p* < 0.001]. Thus, female sex and lower severity of depression, but not trait anxiety or age, predicted higher GM volume in the bilateral thalamus. Entering the cognitive avoidance scale in the second step (β = –0.22) did significantly increase the predictive value of the model [Δ*R*^2^ = 0.04, Δ*p* = 0.02; *F*(6, 107) = 6.32, *p* < 0.001].

### Vigilance

A cluster in the bilateral thalamus exceeded the significance threshold (peak voxel xyz: 5 –18 13, *T* = 4.36, *p* < 0.001, cluster size: 1,532, cluster *p*_FWE_ < 0.001), showing increased GM volume being associated with higher vigilance scores, see Figure 1B. Additionally, a cluster in the left middle temporal gyrus (peak voxel xyz: –50 –16 –9, *T* = 4.24, *p* < 0.001, cluster size: 357, cluster *p*_FWE_ = 0.09) reached marginal significance, indicating a positive correlation (Table 2).

In the first step of hierarchical regression analyses in SPSS, variance in bilateral thalamic GM volume was significantly explained by sex (β = 0.33; *p* < 0.001), with women showing larger volumes, but not by STAI, BAI-T, BDI-II, or age [*p*s > 0.08, *R*^2^ = 0.18; *F*(5, 108) = 4.82, *p* < 0.001]. Female sex predicted heightened GM volume in the bilateral thalamus, but not trait anxiety, depressive symptoms, or age. Entering the vigilance scale in a second step (β = 0.28), significantly increased the predictive value of the model [Δ*R*^2^ = 0.06, Δ*p* = 0.003; *F*(6, 107) = 5.83, *p* < 0.001]. For the marginally significant cluster in the middle temporal gyrus, variance in GM volume was not significantly explained by trait anxiety, depression scores, age, or sex (all *p*s > 0.12), *R*^2^ = 0.07; *F*(5, 108) = 1.66, *p* < 0.15. Entering the vigilance scale in the second step significantly enhanced the explained variance in GM volume [Δ*R*^2^ = 0.13, Δ*p* < 0.001; *F*(6, 107) = 4.55, *p* < 0.001].

### Independent Prediction of Thalamic Gray Matter Volume by Cognitive Avoidance and Vigilance

Bilateral thalamic GM volume was significantly explained by vigilance in the first step of hierarchical regression analyses [β = 0.34; *R*^2^ = 0.11; *F*(1, 112) = 14.44, *p* < 0.001]. Including cognitive avoidance in a second step (β = –0.25) significantly increased the predictive value of the model [Δ*R*^2^ = 0.05, Δ*p* = 0.009; *F*(2, 111) = 11.18, *p* < 0.001].

When entering cognitive avoidance in the first step of the model, the scale significantly predicted bilateral thalamic GM volume [β = –0.31; *R*^2^ = 0.10; *F*(1, 112) = 12.09, *p* < 0.001]. Including vigilance in a second step (β = 0.29) significantly enhanced the explained variance in thalamic GM volume [Δ*R*^2^ = 0.07, Δ*p* = 0.003; *F*(2, 111) = 11.12, *p* < 0.001].

## Discussion

The aim of this study was to investigate the relationship among coping strategies in anxiety-inducing situations and volumetric GM alterations in a non-clinical sample of young adults. Vigilant coping showed a significant positive association with GM density in the thalamus, whereas cognitive avoidance was negatively related to GM volume in this brain region. Despite a moderate correlation between cognitive avoidance and vigilance in our sample, both coping styles independently contributed to the prediction of thalamic GM volume. This is in line with Krohne’s (2) two-dimensional conception of cognitive avoidance and vigilance and a postulated difference in the underlying motivation for each strategy. Whereas cognitive avoidance serves the purpose of reducing emotional arousal and is often employed by individuals with a low tolerance for intense anxiety states, vigilance is assumed to reduce uncertainty in individuals who are especially affected by states of uncertainty.

Our results indicate that healthy individuals with a dispositional tendency to monitor the environment for potential threats, with the aim of being prepared for potential undesirable outcomes, demonstrate volumetric increases in the thalamus. On the other hand, individuals who tend to divert their attention away from distressing internal or external stimuli, to inhibit their further processing, showed reductions in thalamic GM density.

The thalamus is a subcortical brain structure that operates at an early stage in the visual encoding of emotionally salient stimuli (28). The thalamus contributes to the selection of relevant information from the environment and mediates awareness of visual information (29, 30). Activity in the thalamus has also been linked to the processing of emotional scenes (31) and to the anticipation of anxiety-provoking events and pain (32, 33). Additionally, the thalamus appears to be involved in the maintenance of an alert state (34) and in the regulation of vigilance and arousal (35). Given its role in processing emotional stimuli and in mediating attentional processes and alertness, an enlarged thalamus volume may indicate an increased capacity for the maintenance of vigilance in individuals who habitually search for threat-relevant information in their environment. On the other hand, a dispositional tendency to cognitively avoid threat-related information may be associated with a lower capacity for constant vigilance and attention. The thalamus is substantially connected to the amygdala (36) and the prefrontal and tempo-parietal cortex (37), and exerts a strong influence over activity in these brain areas. One might speculate that an enlarged volume reflects an increased (reciprocal) connectivity to brain regions that are implicated in the processing of emotional cues and attentional control. This conclusion is tentative, as the relation between volumetric alterations in the thalamus and the functional consequences remains largely unknown.

According to the MCM (2), highly vigilant individuals are characterized by intolerance for uncertainty about potential threats, a state that is closely linked to the experience of anxiety (38). In our study, trait anxiety showed a moderate positive correlation with vigilant coping and a moderate negative correlation with cognitive avoidance [see also (9)]. Nonetheless, the relationship among coping styles and volumetric alterations in the thalamus remained significant when accounting for a potential influence of trait anxiety. This implicates that, above and beyond a more general anxious temperament, dispositional strategies to deal with aversive events and anxious arousal appear to explain incremental proportions of variance in thalamic GM volume.

Some functional imaging studies pointed to increased reactivity to threat in prefrontal, temporal, or parietal areas in high cognitive avoidance, relative to high vigilance (18–20), but see Leehr et al. (21) for null results in a correlational approach. Increased responsivity in the frontal and temporo-parietal cortex, amygdala, hippocampus, and thalamus have also been reported in sensitizers, compared to repressors, depending on the emotional quality of the stimuli and their ambiguity (16, 19). In line with Leehr et al. (21), we did not find evidence for a relationship between coping styles and morphological alterations in cortical brain areas, with the exception of a marginally significant cluster in the middle temporal gyrus, a brain region that has been implicated in emotion processing [e.g., (31, 39)]. Here, a vigilant coping style was related to greater GM volume. Vigilance and cognitive avoidance have been considered as coping strategies of higher order, which encompass a number of different coping behaviors and cognitions (40). Both concepts include groups of different cognitive functions, such as appraisal, attention orientation, planning, recollection of memories, and self-regulation under threat conditions. These higher cognitive processes have been assigned to widely distributed cortical brain networks, including the dorsolateral, medial, lateral, and orbital prefrontal, anterior cingulate, and parietal cortex [e.g., (17, 41–43)]. One might argue that different coping behaviors and cognitions relevant for cognitive avoidance and vigilance cannot be precisely mapped to a specific cortical brain area. There may be no basic relay systems on a cortical level involved in all cognitive avoidant/vigilant strategies. Krohne’s broad definition of coping styles might mask structural alterations in cortical regions that are limited to only one specific coping behavior or cognition. This assumption is partly corroborated by findings from the above-mentioned functional neuroimaging research. Across these studies, there is little overlap between the specific cortical regions activated as a function of repression or sensitization.

In general, findings on brain structural and functional correlates of anxiety-related coping remain heterogeneous and point to the importance of further research. In our study, only healthy and young participants were included. Future studies are needed to replicate our findings and to investigate brain structural correlates of coping styles in clinical and older populations. In addition, to overcome a potential problem with the moderate overlap between cognitive avoidance and vigilance, in future studies subjects may be divided into high and low scorers on each dimension, resulting in four coping style groups for the investigation of differences in GM volume.

It appears worthwhile to further investigate the structural and functional neural substrates of cognitive coping strategies since cognitive avoidance and vigilance can have important implications for mental health and wellbeing. It has been argued that repressive coping could constitute a rather maladaptive strategy to deal with dangerous or aversive events [e.g., (44)]. Denial or attentional diversion from potential threats and problems, which require a solution, may undermine adaptive problem-focused approaches, and may lead to an enduring exposure to challenging environments. However, Coifman et al. (45) have demonstrated a protective function of repressive coping with respect to psychological health, particularly after the experience of extreme adverse life events. Indeed, cognitive avoidance appears to be associated with lower neuroticism, negative affectivity, and anxiety, whereas vigilant coping shows the opposite pattern (4, 9). Constant vigilance and facilitated threat-detection may increase the perception of potential harm (38), and therefore, may intensify the experience of anxiety and negative affect [see also (40)]. However, increased vigilance lowers the probability of being confronted with aversive events without preparation. Interestingly, a protective effect of high vigilance on the experience of stress in high-risk situations has been reported (46). The effectiveness of anxiety-related coping strategies appears to depend on the presence of actual threats and to the degree to which they can be controlled. Thus, previous research does not allow an unambiguous assignment of cognitive avoidance and vigilance as clearly adaptive or maladaptive coping strategies.

Our data provide evidence that the gray matter volume in the thalamus varies as a function of dispositional preferences for strategies to deal with anxiety-provoking situations. Volumetric variability in the thalamus was additionally explained by female sex and severity of depressive symptoms but was no mere correlate of a more general disposition to experience anxiety in novel or ambiguous situations, i.e., trait anxiety. It has been found previously that women have larger gray matter volumes in the bilateral thalami [see (47), for a meta-analysis of sex differences in human brain structure]. Additionally, we found sex differences in cognitive avoidance, with women using cognitive avoidant strategies less frequently than men. This finding is in line with earlier studies [e.g., (4, 9, 12)]. It has been argued that men may be more socialized to cover up their emotions, and therefore, may be more likely to rely on coping strategies as denial and avoidance (48). Krohne et al. (4) suggested that sex differences in coping styles may be explained by sex differences in the perception of physiological sensations and in the use of internal or external sources of information for the determination of emotional states [see (49)]. One might also speculate that volumetric increases in the thalamus in women, compared to men, could be a prerequisite for a lower dispositional tendency to use cognitive avoidance as a coping strategy. Further research is required to clarify these issues.

Our finding that higher severity of depression goes along with lower gray matter volume in healthy individuals is consistent with and expands results from prior research indicating lower thalamic volume in patients with clinical depression (50) and in subclinical individuals suffering from mild depressive symptoms (51).

The cross-sectional nature of our study prevents us from speculating about the direction of the effects. Volumetric alterations in the thalamus may underlie neurobiological mechanisms in the manifestation of inter-individual differences in coping strategies. However, they may also be the long-term consequences of down-regulated attention in individuals who preferably employ cognitive avoidance in their daily lives and may result from continuous attempts to watch out for potentially threatening information in the environment in vigilant individuals.

## Data Availability Statement

The raw data supporting the conclusions of this article will be made available by the authors, without undue reservation.

## Ethics Statement

The studies involving human participants were reviewed and approved by the Ethics Committee at the University of Leipzig, Medical Faculty. The patients/participants provided their written informed consent to participate in this study.

## Author Contributions

VG, TS, DL, AK, K-TH, CB, and BE conceived and designed the experiment. VG, SJ, CW, AB, and SM were involved in recruitment of participants and data collection. VG, DL, and TS outlined the manuscript. VG analyzed the data and wrote the first draft of the manuscript. All authors contributed to the article and approved the submitted version.

## Conflict of Interest

The authors declare that the research was conducted in the absence of any commercial or financial relationships that could be construed as a potential conflict of interest.

## Publisher’s Note

All claims expressed in this article are solely those of the authors and do not necessarily represent those of their affiliated organizations, or those of the publisher, the editors and the reviewers. Any product that may be evaluated in this article, or claim that may be made by its manufacturer, is not guaranteed or endorsed by the publisher.

## Footnotes

1
http://dbm.neuro.uni-jena.de/vbm
